# Drug-Coated Balloons in Coronary Bifurcation Disease: A State-of-the-Art Review

**DOI:** 10.3390/jpm16020075

**Published:** 2026-01-31

**Authors:** Saad M. Ezad, Natasha Khullar, Peter O’Kane, Jonathan Hinton

**Affiliations:** 1 Dorset Heart Centre, Royal Bournemouth Hospital, Castle Lane East, Bournemouth BH7 7DW, UKnatasha.khullar@nhs.net (N.K.); peter.okane@nhs.net (P.O.); 2 British Heart Foundation Centre of Research Excellence, School of Cardiovascular and Metabolic Medicine and Sciences, King’s College London, London SE1 7EH, UK

**Keywords:** coronary bifurcation, drug-coated balloons, DCB, PCI, coronary artery disease

## Abstract

Coronary bifurcation disease remains one of the more challenging lesion subsets to treat with percutaneous coronary intervention due to bifurcation geometry and increased risk of target lesion failure. Whilst a provisional approach is preferred in most bifurcations, two-stent techniques may be required where there is a high risk of side branch compromise or a bailout; however, this further increases procedure complexity. Drug-coated balloons (DCBs) are emerging as a promising alternative that allow vessel healing without leaving behind a permanent metallic implant by delivering antiproliferative medication directly to the vessel wall and simplifying procedures. This state-of-the-art review summarises the current evidence and the evolving role of DCBs in the management of coronary bifurcation lesions with a focus on patient- and lesion-specific factors that might influence the treatment strategy choice.

## 1. Introduction

Drug-coated balloons (DCBs) deliver antiproliferative drugs directly to the vessel wall and may overcome limitations such as early restenosis associated with plain old balloon angioplasty (POBA), first described by Andreas Gruntzig in 1977 [1]. The development of bare metal stents (BMSs) was the initial step in the attempts to overcome such limitations with the landmark BENESTENT and STRESS trials demonstrating the superiority of BMSs over POBA in reducing restenosis and repeat revascularization [2,3]. However, neointimal hyperplasia and in-stent restenosis (ISR) then emerged as significant challenges, leading to the development of drug-eluting stents (DESs) coated with antiproliferative drugs.

Second-generation DESs have substantially improved outcomes compared with earlier devices, with lower rates of ISR and stent thrombosis. However, the persistent risk of target lesion failure (TLF), estimated at ~1–2% annually in contemporary trials [4], underscores that permanent metallic scaffolds are not a panacea. These challenges have renewed enthusiasm for the ‘leave nothing behind’ concept, in which DCBs deliver an antiproliferative drug directly to the vessel wall without leaving a scaffold. This approach has already demonstrated efficacy in ISR and small vessel disease [5,6,7,8,9,10]; however, its potential role in large vessel disease and more complex disease subsets remains unclear.

Bifurcation lesions, present in approximately 15–20% of all percutaneous coronary intervention (PCI) procedures [11], remain among the most technically challenging scenarios in interventional cardiology. Discrepancies in vessel size between the main and side branches, plaque, or carina shift compromising side branch flow, and the need for complex multi-stent techniques contribute to procedural difficulty. Registry and randomised trial data consistently demonstrate that bifurcation PCI is associated with higher rates of restenosis, stent thrombosis, and target lesion revascularisation (TLR), particularly at side branch ostia, compared with non-bifurcation lesions [12,13,14]. Furthermore, complex bifurcation strategies are associated with longer procedural times, increased radiation, contrast exposure, and outcomes that are closely linked to both operator experience and procedural volume [15,16].

In true bifurcation lesions, where both the main and side branches are significantly diseased, two-stent techniques such as culotte or double kissing (DK) crush can achieve satisfactory angiographic results [17] but often at the cost of increased technical complexity, overlapping metal layers, and higher risks of restenosis and stent thrombosis at the carina [18,19]. In contrast, a DCB-based approach offers a simplified strategy that preserves bifurcation geometry and minimises metallic burden. DCBs have the potential to reduce procedural time, radiation exposure, and the need for prolonged antiplatelet therapy by obliviating strut distortion, incomplete ostial coverage, and multiple stent layers as well as uneven drug delivery [20].

This review provides a comprehensive analysis of the current evidence and evolving strategies regarding the use of DCBs in de novo coronary bifurcation disease, with a specific focus on the patient and lesion characteristics that guide clinical decision-making. We examine the rationale, technical considerations, patient factors, and clinical data supporting DCB-based approaches, highlighting their potential role in redefining bifurcation PCI.

## 2. Bifurcation Disease and Traditional PCI Strategies

**Figure 1:**
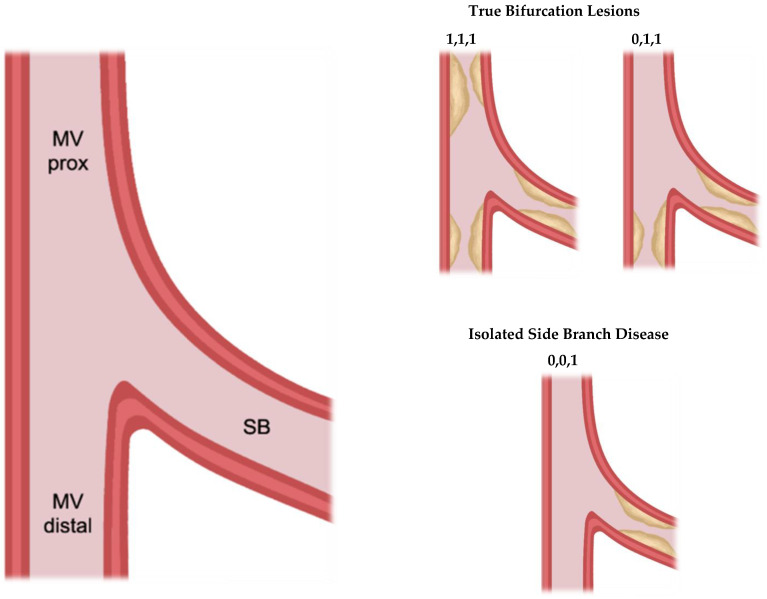
Medina classification for coronary bifurcation lesions. Each bifurcation is assigned a three-digit binary code, and each digit corresponds to a specific anatomical segment: the proximal main vessel (MV prox), distal main vessel (MV distal), and side branch (SB). A value of ‘1’ denotes ≥50% stenosis, while ‘0’ indicates <50% stenosis. Representative examples of true bifurcation lesions and isolated side branch disease are illustrated.

Bifurcation lesions (defined by the anatomical SYNTAX score as ≥50% diameter stenoses occurring within 3 mm of a significant division of a major epicardial coronary artery) are particularly prone to atherosclerosis due to the complex interplay between vascular anatomy and haemodynamics [21]. Within bifurcations, the carina itself is relatively protected by high, unidirectional shear stress, whereas the lateral walls opposite the carina experience low and oscillatory shear stress, promoting lipid disposition and inflammation [22]. This results in a non-uniform distribution of plaque, with lesions typically forming along the lateral walls [23]. Bifurcations are classically described using the Medina classification, based on the presence of plaque in the proximal and distal main vessel and the side branch [24] (Figure 1). True bifurcation lesions are those where atherosclerosis extends into both daughter branches (Medina 1,1,1 or 0,1,1). However, the definition of which side branch warrants intervention remains debated, as angiographic stenosis often over-estimates the physiological significance of a side branch lesion [25]. Current international guidelines favour a provisional philosophy, in which the main vessel is stented first and the need for potential intervention to the side branch re-assessed afterwards [26]. While effective, this approach can limit the choice of bifurcation stenting techniques, particularly precluding the use of the DK crush strategy.

Side branch compromise, typically caused by carina or plaque shift, can occur in up to 8% of cases with a provisional approach and is associated with an increased risk of death and/or myocardial infarction [27]. Plaque shift is associated with a large plaque burden in the proximal main vessel, whilst carina shift tends to occur when the stent is oversized for the distal main vessel and is the more prominent reason for side branch compromise [28]. Conversely, it is plaque shift which is correlated with a physiologically significant side branch stenosis following provisional stenting; however, only 15% of side branches with a post-stenting angiographic diameter stenosis of ≤50% will be physiologically significant [29]. A postulated third mechanism of side branch compromise following DES implantation is ostial deformation leading to an eclipse-shaped ostium [30].

From a patient perspective, identifying upfront a side branch at risk of compromise is highly advantageous as this informs procedural planning potentially supporting a two-stent strategy. A two-stent strategy, however, introduces procedural complexity, resulting in more contrast/radiation and a longer procedure. Real-world registry data suggest higher rates of TLR with two-stent strategies, while randomised data remain discrepant (Table 1) [31]. In the DKCRUSH V trial, which enrolled patients with complex true left main bifurcation disease, the DK crush technique significantly reduced the composite endpoint of target lesion failure compared with provisional stenting (PS) at 1 year (5.0% DK crush vs. 10.7% PS, *p* = 0.02), driven by lower rates of myocardial infarction and TLR (3.8% vs. 7.9%, *p* = 0.06) [17,32]. Similarly, the DEFINITION II trial demonstrated that in angiographically complex bifurcations, systematic two-stent strategies (predominantly DK crush) reduced 12-month target lesion failure (6.1% vs. 11.4% PS, *p* = 0.019) and TLR (2.4% vs. 5.5% PS, *p* = 0.049) compared with provisional stenting [33]. By contrast, the EBC MAIN trial, which randomised patients with true left main bifurcations to a provisional approach or routine two-stent strategies (mainly culotte, T, or TAP), found no significant difference in the composite of death, myocardial infarction, or TLR at 12 months (14.7% PS vs. 17.7% two-stent, *p* = 0.34) [16]. These divergent results, reflecting differences in lesion complexity, stenting techniques, and operator experience, underscore the need for a personalised and patient- and lesion-specific revascularisation approach.

**Figure 2:**
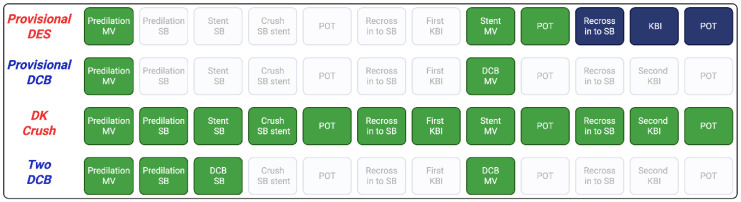
Steps involved in DCB vs. DES bifurcation techniques. Blue = optional, green = mandatory.

Even when following the provisional approach, main vessel stenting can be associated with adverse long-term outcomes, including restenosis and thrombosis, with uncovered struts at the carina as a potential contributing factor [23]. A balloon-only or hybrid strategy using DCBs for the side branch offers a compelling alternative by avoiding carina or plaque shift, reducing procedural steps compared with two-stent techniques (Figure 2), and eliminating the long-term risks associated with a permanent metallic scaffold.

In clinical practice, the bifurcation strategy should be tailored to lesion anatomy and patient-specific factors. Simple (non-true) bifurcations are best treated with a provisional stenting approach, whereas complex true bifurcations—meeting DEFINITION II criteria—generally merit consideration of an upfront two-stent technique. As discussed above, DCBs may offer an attractive alternative in the side branch, particularly in patients at high bleeding risk, by potentially shortening the dual antiplatelet therapy duration, although this remains to be validated in bifurcation-specific randomised trials. The choice between DCB and DES should integrate these anatomical and clinical considerations in addition to the anticipated procedural tolerance. Smaller vessels, frail comorbid patients, renal dysfunction, and limited ventricular reserve favour the use of DCBs to simplify and shorten procedures and reduce the risk of restenosis.

## 3. Drug-Coated Balloon Technology Overview

The concept of DCBs emerged in the early 2000s following preclinical evidence demonstrating that paclitaxel effectively inhibits neointimal proliferation in animal models post-balloon angioplasty [43]. Unlike DESs, which provide sustained drug delivery via a polymeric coating and metallic scaffold, DCBs enable transient, localised delivery of an antiproliferative agent without leaving a permanent implant. This ‘leave nothing behind’ approach has potential advantages for specific lesion subsets and patient factors, such as small-calibre vessels, bifurcation lesions, diffuse distal disease, ISR, or patients with high bleeding risk. In these settings, DCBs have demonstrated non-inferior outcomes compared to contemporary DESs in terms of major adverse cardiac events (MACEs), TLR, and late lumen loss (LLL), while allowing for shorter durations of dual antiplatelet therapy [5,6,7,8,9,10,16,44,45,46].

A typical DCB consists of a semi-compliant angioplasty balloon coated with an antiproliferative drug, most commonly paclitaxel or sirolimus, embedded within an excipient or carrier matrix. The coating is designed to remain stable during catheter tracking and to facilitate rapid, homogenous drug transfer to the vessel wall during balloon inflation (typically 30–60 s), ensuring sufficient tissue retention to inhibit smooth muscle cell proliferation and suppress neointimal hyperplasia [43,47]. The excipient plays a pivotal role in maintaining coating integrity and modulating drug release kinetics at the target site [48,49,50]. Its physicochemical properties influence coating morphology, adhesion, drug solubility, and interaction with the arterial wall, thereby affecting clinical performance and safety [48,50,51,52]. Urea-based formulations (e.g., IN.PACT Admiral) facilitate rapid drug release and efficient tissue uptake but may be more prone to particulate loss [50,53]; butyryl-trihexyl citrate (BTHC)-based excipients (e.g., Lutonix) enhance coating stability and reduce premature drug loss, though with slower drug release kinetics [47,51]; and shellac-based systems (e.g., Genoss DCB) provide a balanced profile of coating durability and controlled release, with emerging data indicating non-inferiority to urea-based devices [50,53]. Collectively, these excipient-dependent differences underscore the absence of a class effect among DCBs, mandating device-specific assessment of efficacy and safety [20,47]. Furthermore, differences in deliverability across devices require careful consideration of vessel tortuosity and compliance when selecting the optimal DCB for individual patients.

## 4. Paclitaxel- and Limus-Coated DCBs

DCBs are differentiated by the antiproliferative drug employed—either paclitaxel or limus agents (e.g., sirolimus, everolimus, zotarolimus, and biolimus). While both paclitaxel-coated balloons (PCBs) and limus-coated balloons are designed to deliver antiproliferative drugs to the vessel wall during balloon inflation, their device compositions and pharmacologic properties differ significantly (Table 2). Thus, it is important to consider these factors when making a choice of DCB to tailor it to the lesion and specific needs of the patient.

PCBs use paclitaxel, a highly lipophilic, cytotoxic agent that irreversibly binds to the β subunit of tubulin, inhibiting microtubule disassembly and cell-cycle progression [47,54]. This property allows for rapid and efficient drug transfer and retention in the vessel wall after a short contact time. The drug is typically applied to the balloon surface in a crystalline or amorphous form, often with an excipient such as iopromide or urea [55]. Crystalline coatings provide higher stability and tissue retention but may increase downstream particulate generation. Typical paclitaxel doses on the balloon range from 2 to 3.5 μg/mm^2^, although newer-generation devices are exploring higher dosing strategies to improve tissue uptake [47,54].

Limus-coated balloons deliver sirolimus or its analogues and exert their therapeutic effect primarily through inhibition of the mammalian target of rapamycin (mTOR) pathway [56]. Upon delivery to the vessel wall, sirolimus forms a complex with FKBP12, which then binds to and inhibits mTOR, a serine/threonine kinase central to cell-cycle regulation. This inhibition blocks vascular smooth muscle cell (VSMC) progression from the G1 to S phase, thereby suppressing proliferation and neointimal hyperplasia after angioplasty [47,56,57].

Unlike paclitaxel, limus agents are less lipophilic, making efficient vessel wall transfer and retention more challenging. To address this limitation, limus DCBs often employ advanced excipient systems (e.g., phospholipids, microgels, or crystalline formulations) to enhance drug delivery and prolong tissue residence time. Crystalline sirolimus coatings have demonstrated improved stability and retention compared to amorphous forms, but overall tissue uptake remains lower than with paclitaxel [58]. Everolimus-coated balloons have also been developed using microgel-based excipients to improve sustained release and reduce drug loss during delivery [59]. Among newer technologies, the SELUTION SLR balloon incorporates biodegradable polymer (Poly-lactic-co-glycolic acid—PLGA) micro-reservoirs within a lipid-based adherent matrix, enabling controlled sirolimus release and improving vessel wall retention compared with earlier sirolimus-coated balloon (SCB) designs [60,61,62].

In clinical practice, there is variance in the performance of different DCBs with some showing similar performance but others showing variation in rates of TLF, MACEs, and TLR at 9–12 months [42,60,62,63,64,65,66,67,68]. In ISR specifically, there is variation in the data with some showing similar performance whilst others suggest superiority of PCBs over limus devices [64,67]. Emerging SCB technologies, including nano-encapsulated and crystalline formulations, have demonstrated promising mid- and long-term safety, with no increase in mortality or adverse events relative to PCBs [42,63,69,70,71,72,73]. Overall, the choice between PCBs and SCBs may be influenced by pharmacokinetics, but currently each DCB needs to be considered individually and even within the specific drug types (limus versus paclitaxel), each DCB is likely to perform differently due to the significant variations in technology and design.

## 5. Drug Transfer Dynamics

The efficiency of drug delivery in DCB therapy is influenced by several interdependent factors, including coating microstructure, excipient properties, balloon–vessel contact pressure, inflation time, and vessel wall characteristics. Only a fraction of the loaded drug is ultimately transferred to the vessel wall, with significant variation between DCBs. Substantial losses are possible during device handling and transit, and once delivered, the remainder can be rapidly cleared by blood flow if not retained in the tissue [48,74,75]. Uniform balloon apposition is critical; irregular contact due to vessel anatomy, plaque burden, or balloon folding can result in heterogeneous drug distribution [75,76]. Furthermore, the micro-morphology of the coating (e.g., crystalline, amorphous, or microneedle structures) influences not only the surface area for transfer but also the local pressure, both of which affect tissue penetration and retention [48,50,75].

Procedural strategies also influence drug transfer dynamics. For example, kissing balloon inflation (KBI), if employed in bifurcation interventions, may disrupt the freshly deposited drug layer if performed immediately after DCB deployment. This could result in drug washout or redistribution before adequate tissue uptake occurs [75,77]. Both computational and experimental data suggest that additional balloon inflations post-DCB can transiently reduce local drug concentrations, although these differences tend to diminish over time (within minutes) [77]. Despite these considerations, findings from the DCB-BIF trial indicate that KBI following a side branch PCB is not associated with adverse outcomes, suggesting that post-PCB manipulation is acceptable when clinically necessary, though it remains advisable to minimise additional intervention whenever possible [40]. These data again highlight the challenge of adopting a universal treatment strategy; instead, clinicians should adopt a patient- and lesion-focused approach whilst also taking into account the individual properties of the specific DCB.

## 6. DCB Use in Bifurcation Lesions

### 6.1. Provisional Main Vessel Treatment

Bifurcation lesions where the side branch does not require upfront treatment may be simplified by using a DCB alone in the main vessel, thereby avoiding the complexities of kissing balloon inflation or proximal optimisation (Figure 2). Moreover, the use of a DCB as opposed to a DES in the main vessel also appears beneficial for the side branch ostium with luminal gain and a reduction in diameter stenosis at the 6–9 month angiographic follow up observed [78]. These findings have been confirmed with the use of OCT 9 months following main vessel treatment with a paclitaxel DCB: a median ostial luminal area gain of 52.1% was observed in the SB [79].

However, caution is warranted, as highlighted by the REC-CAGEFREE I trial, which randomised 2272 patients with de novo, non-complex coronary artery lesions to receive either paclitaxel-coated balloon angioplasty with the option of rescue stenting or DES. The DCB group did not meet the non-inferiority criteria, showing a higher incidence of the composite primary outcome (cardiovascular death, target vessel myocardial infarction, and clinically indicated target vessel revascularisation; 2.3 vs. 1.2%, *p* = 0.054) with a trend to higher CV death at two years [41]. Interestingly, this study randomised participants after vessel preparation, which likely resulted in significantly higher use of specialty balloons (scoring/cutting) than is commonly seen in DES PCI. This may have favoured DES PCI by encouraging more fastidious lesion preparation than is currently performed in routine clinical practice. Notably, a significant interaction with vessel size was observed: the DCB fared worse in larger vessels (HR 3.04; 95% CI 1.72–5.38), which has important implications for DCB use in the main branch of a bifurcation. In the bifurcation lesion subgroup, however, the trial showed equipoise in DCB and DES outcomes (HR 1.25; 95% CI 0.70–2.23), which should be regarded as hypothesis-generating and warranting further study. The combination of these data suggest that (a) provisional DCB treatment in bifurcation is reasonable in main vessels <3.0 mm in diameter and (b) a DCB in a side branch of <3.0 mm is a reasonable strategy for side branch management during main vessel DES PCI. When considering the results of REC-CAGEFREE, it is important to highlight that the DCB utilised in this study is not available outside China and there are limited prior data on its efficacy at drug delivery. This highlights the importance of not considering DCBs as having a class effect; their performance varies significantly and whilst REC-CAGFREE showed that this particular PCB was inferior to a DES, it is likely that other PCBs will perform differently (whether that be better or worse).

The recently presented SELUTION DeNovo trial randomised 3326 patients with de novo lesions in 2.0–5.0 mm vessels, excluding only acute coronary syndromes requiring urgent intervention, graft disease, CTOs, and ISR. At one year, the primary endpoint of target vessel failure (a composite of cardiac death, target vessel revascularization, and target vessel myocardial infarction) occurred in 4.4% in the DES arm and 5.3% in the DCB arm, meeting the prespecified variable non-inferiority margin (*p* = 0.02) [42,60]. In contrast to REC-CAGEFREE, randomisation occurred before lesion preparation, resulting in greater use of specialty balloons in the DCB arm (28.5% vs. 7.9%). Bifurcation lesions accounted for 32.1% of the DCB cohort; exploratory subgroup analysis showed no significant difference in outcomes (absolute risk difference 0.23%; 95% CI –2.49 to 2.94). These data would support the use of DCBs for main vessel destination therapy as a reasonable alternative to DES implantation. It is essential to highlight that the data from SELUTION DeNovo only apply to the selution balloon; there are significant variances in the design and performance of different DCBs, even those using sirolimus, and therefore, a class effect cannot be inferred [61,62]. Longer-term follow up at five years will be extremely important to evaluate whether a DCB strategy results in improved longer-term outcomes [60].

### 6.2. Isolated Side Branch Disease

Medina 0,0,1 lesions represent the least prevalent bifurcation subtype; however, these have been associated with the highest rates of adverse outcomes following treatment with DES [80] and, therefore, may be the most attractive scenario for DCB use. The PEPCAD-BIF trial was the first trial to randomise participants to a DCB-only approach. A total of 64 patients were assigned to POBA or DCB application following lesion preparation for Medina 0,1,1; 0,1,0; or 0,0,1 bifurcations with a mean vessel diameter of 2.4 mm; at the 9-month follow up, less late lumen loss was observed in the DCB group (0.13 vs. 0.51 mm, *p* = 0.013) [34]. Given these findings DCB use may be the preferred strategy in such lesions where the side branch is ≤3 mm. In side branches over 3 mm in diameter, operators should consider patient factors, particularly bleeding risk and the need for dual antiplatelet therapy. Most importantly, however, discussion with the patient is essential to ensure that patient preferences are considered when deciding upon whether to use a DCB or DES.

### 6.3. Full DCB Treatment of Both Main Vessel and Side Branch

To date, no randomised data for a DCB-only strategy in the main vessel and side branch are reported; therefore, evidence is limited to case series and single-centre reports. Schulz et al. reported outcomes on 39 consecutive cases including LMS bifurcation lesions with follow up angiography at 4 months. No MACEs were reported, whilst three patients demonstrated restenosis, all of which involved the LMS [35]. Bruch et al. compared 127 patients, of whom 70 received DCB-only treatment and the remainder were treated with a hybrid strategy, resulting in comparable TLR (4.5% vs. 3.6%, *p* = 0.80) and MACE (6.1% vs. 7.3%, *p* = 0.79) rates at 9 months [36]. Kitani et al. evaluated the application of DCBs following directional coronary atherectomy in 129 patients, the majority of whom had left main bifurcation disease, finding a TLR rate of 3.1% at 12 months [37].

Whilst these observational data are promising, further randomised studies are required before a full DCB approach to bifurcations can be considered the standard choice for bifurcation management. The EBC-DCB (NCT06822322) trial will randomise 750 participants to a stepwise provisional DES strategy with kissing balloon inflation or a DCB strategy with DCB use in both the side branch and main vessel. Pending data that show DCBs have similar performance to DES in this situation, a DES should remain the standard choice unless there are specific features that favour a DCB approach, such as complex bifurcation anatomy.

### 6.4. Hybrid Strategy

A common strategy to simplify bifurcation procedures has been to use a POBA strategy for the side branch whilst employing a provisional DES approach to the main vessel. Utilising a DCB in place of the POBA in the side branch in a so-called hybrid strategy has been associated with less late lumen loss and reduced TLR and MACEs [81,82]. Reassuringly, these findings have been replicated in LMS bifurcations [83]. A sub-study of the HYPER trial involving 50 patients with true bifurcation disease reported a procedural success rate of 96% and favourable short-term outcomes at 12 months [39]. It is important to consider each individual patient/lesion to decide on the appropriate strategy because bifurcation lesions with long (>10 mm) side branch disease are associated with a higher rate of target lesion revascularisation following application of a paclitaxel-coated balloon [84].

The BEYOND study, conducted at 10 centres with 222 participants in China, was the first reported randomised comparison of paclitaxel DCBs compared to POBA of the SB in a non-left main bifurcation. The primary outcomes of angiographic SB restenosis at 9 months occurred less frequently in the DCB group (target lesion stenosis (TLS) 28.7% in DCBs vs. 40.0% in POBA, *p* < 0.0001) with lower late lumen loss (−0.06 ± 0.32 mm vs. 0.18 ± 0.34 mm in POBA, *p* < 0.0001) [38]. The rate of clinical events, including non-fatal myocardial infarction, was similar, although follow up was relatively short. The DCB-BIF trial randomised 784 patients with a residual SB stenosis of ≥70% after implant of DES to the main vessel to either paclitaxel DCB or POBA, with both groups undergoing final KBI after SB intervention [40]. At the 1-year follow up, the composite primary endpoint (cardiac death, target vessel myocardial infarction, or clinically driven target vessel revascularisation) occurred less frequently in the DCB group (HR: 0.56; 95% CI: 0.35–0.88) [40]. Interestingly, the difference was largely driven by myocardial infarction occurring predominantly within the first two days, suggesting that DCBs may confer an acute protective effect beyond long-term restenosis prevention. The mechanism underlying this observed reduction in myocardial infarction is uncertain and likely multifactorial; it may result from a combination of meticulous lesion preparation, optimal balloon sizing, longer inflation time of the DCB compared with POBA, and potential early biological effects of drug delivery that mitigate acute thrombus formation.

Similarly, the OCVC BIF study, recently presented at TCT, randomised patients with disease involving a bifurcation with a side branch between 2 and 3.0 mm in diameter to either a DCB (SeQuent Please, paclitaxel balloon) or no further treatment after a main vessel DES, followed by KBI and POT. The primary endpoint was side branch vessel stenosis on coronary angiography at 9 months (using quantitative coronary angiography) [85]. There was a significantly reduced side branch restenosis (>50%) in the DCB group (8.1% vs. 18.3% *p* = 0.012) [86]. There were no statistically significant differences in clinical outcomes at one year, although there was a trend to reduced clinically driven target lesion revascularisation in the DCB group (0.7% vs. 3.4% (*p* = 0.20) [86]. These two studies strongly support the use of paclitaxel DCBs for management of a side branch during provisional DES PCI to the main vessel. Interestingly, the main difference between these two studies is that DCB BIF mandated KBI after PCB use in the side branch, whereas OCVC BIF left this at the operator’s discretion. Whilst only a minor point, it is possible that a side branch DCB after main vessel DES implant without subsequent KBI could result in deformation of the main vessel stent, which may lead to less favourable outcomes in the long term. One potential simplification is the use of a DCB in the side branch before a DES in the main vessel. This negates the need for KBI as the DCB will not alter the stent geometry. Furthermore, the passage of a DCB through the side of a main vessel stent could result in loss of drug from the DCB. Further data are therefore required on the optimal order of DCB for the side branch during provisional main vessel DES implantation.

No randomised trials have compared sirolimus DCBs to POBA for side branch disease; the SPACIOUS trial randomised 230 patients to either a sirolimus or paclitaxel DCB, finding the sirolimus DCB to be non-inferior with respect to diameter stenosis in the SB ostium at the 9-month angiographic follow up [87].

## 7. Technical and Procedural Considerations

### 7.1. Lesion Preparation

Lesion preparation is essential to achieve durable long-term results with the use of a DCB because the minimum luminal diameter is strongly associated with angiographic results at follow up [88]. Whilst leaving dissections is a source of anxiety for many interventionalists, the absence of medial dissection on OCT prior to the application of a DCB is the strongest predictor of target lesion failure (HR 8.24, 95% CI 3.15–21.6) [89]. Moreover, medial dissection has been associated with late lumen enlargement following DCB application [90]. Optimal lesion preparation is therefore essential to obtain a durable result, particularly in the case of calcified coronary disease. Advanced calcium modification devices may be required and despite concerns about side branch compromise in bifurcation disease, there is accumulating evidence regarding their safety and efficacy.

Case series have demonstrated the feasibility of using rotational atherectomy (RA) in both the main vessel and side branch without acute side branch closure or perforation [91]. In a sub-study of the PREPARE-CALC trial, use of rotational atherectomy was associated with less side branch compromise as compared to scoring or cutting balloon, predominantly driven by a lower rate of major dissection (2 vs. 13%, *p* = 0.02) [92] findings, which is supported by observational studies [93]. DCBs may be a particularly attractive strategy where treatment of the SB is planned following RA, as two-stent techniques in this scenario are associated with higher rates of revascularisation after 12 months, reflecting the vulnerability of stents in calcific coronary disease [94].

A mechanistic study using femoral arteries from a swine model demonstrated comparable paclitaxel tissue levels when comparing a DCB-only strategy to orbital atherectomy followed by a DCB without evidence of vascular necrosis or delayed healing [95]. Limited data exist with respect to bifurcation lesions and orbital atherectomy; a small 30-patient study found 40% of cases had side branch compromise [96]. Importantly, this was following implantation of the main vessel stent and is therefore unlikely to be as a direct result of orbital atherectomy. In a propensity-matched cohort, 363 patients who underwent orbital atherectomy at a bifurcation lesion had comparable 30-day clinical outcomes to non-bifurcation lesions [97]. DCB use compared to a DES following orbital atherectomy was associated with a smaller post-procedural minimal lumen area (3.83 vs. 4.86 mm^2^, *p* < 0.001); however, 1-year clinical outcomes were comparable and the DCB group had less late lumen loss, demonstrating the feasibility of this strategy [98].

Intra-vascular lithotripsy (IVL) achieved comparable technical success and 1-year MACE rates in bifurcation vs. non-bifurcation lesions with drug-eluting stents [99]. Scarce data exist with respect to the combination of a DCB and IVL, with small case series demonstrating satisfactory procedural success and short-term clinical outcomes [100,101]. The Shockwave IVL + DCB (NCT05625997) trial will evaluate whether IVL has the potential to enhance the results of coronary balloon dilatation of small vessels with calcified lesions, thereby facilitating optimal DCB intervention. Further data are required to help guide clinicians regarding the optimal preparation of calcified lesions prior to DCB intervention.

The choice of preparation strategy needs to be carefully considered based on both an individual patient and lesion basis. From a lesion perspective, the presence and distribution of calcium within the bifurcation should be carefully evaluated and this must be balanced with specific patient characteristics associated with adverse outcomes from calcium modification, such as advanced age. Anatomical features also influence the preferred treatment pathway following preparation. In routine practice, the main vessel provisional stent strategy with selective side branch DCB is supported by extensive evidence and aligns with contemporary EBC and DCB consensus recommendations [20,26]. In anatomies with a wide side branch take-off angle, the risk of carina shift is reduced, and side branch preservation is more predictable [102]. A small distal branch further favours a simplified approach, consistent with guidance discouraging routine stenting in small-calibre vessels [5,26]. In the presence of a calcified carina, limiting metal and maintaining wire access are advantageous [103].

#### 7.1.1. ‘Leave Nothing Behind’ Approach

**Figure 3:**
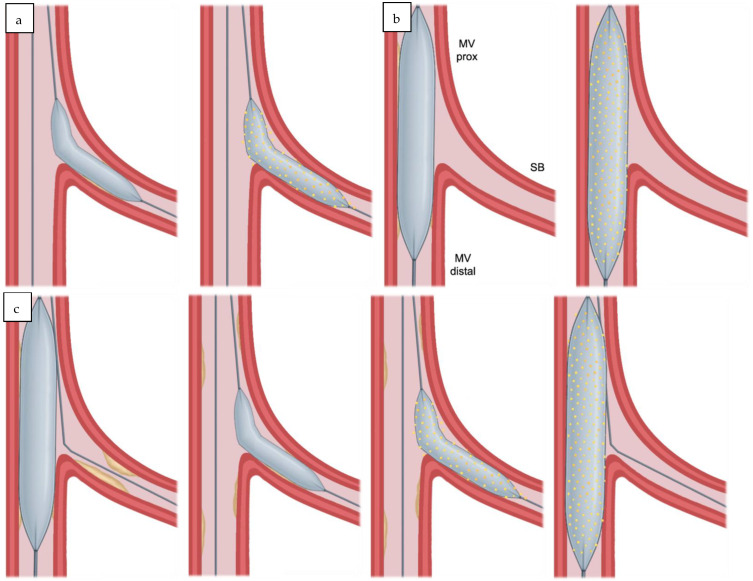

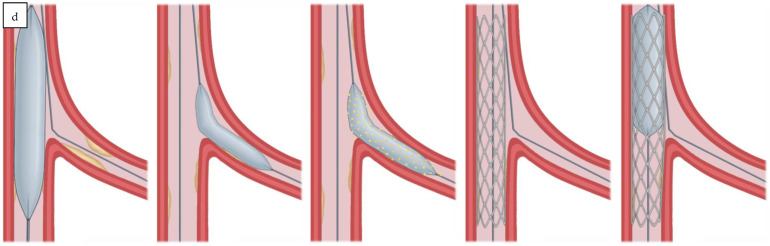
(**a**). Provisional DCB. Pre-dilate MV; DCB MV. (**b**). Isolated SB disease. Pre-dilate SB; DCB SB. (**c**). Two-DCB approach. Pre-dilate MV; pre-dilate SB; DCB SB; DCB MV. (**d**). Hybrid strategy. Pre-dilate MV; pre-dilate SB; DCB SB; stent MV; POT MV.

A full drug-coated balloon strategy in coronary bifurcation disease can be applied in several ways: as a provisional approach to the main vessel, for isolated side branch disease, or to treat both limbs of the bifurcation (Figure 3). Strategy selection is guided by branch morphology, plaque distribution, and vessel dimensions. Procedural planning, including the sequence of branch treatment, balloon sizing, and inflation technique, is essential to achieve optimal luminal gain while limiting the risk of the vessel threatening dissection or plaque shift. Technical considerations for each strategy are outlined below.

#### 7.1.2. Provisional Strategy

For most bifurcation lesions, a provisional approach should remain the default. Careful main vessel preparation is critical and should be guided by intravascular imaging, with the use of specialty balloons or advanced calcium modification devices as required. Following preparation, the angiographic result should be reassessed to determine the final strategy (DES versus DCB). A personalised approach to the final strategy should include assessment of specific patient factors such as age, presence of diabetes, and bleeding risk associated with dual antiplatelet therapy.

Dissections occur in up to 40% of cases after adequate lesion preparation [104]. Nevertheless, a DCB approach can be safely pursued if the following criteria are satisfied: (i) preserved TIMI III flow, (ii) absence of flow-limiting dissection or contrast staining, (iii) residual stenosis ≤30% [20].

The DCB should be sized 1:1 to the distal main vessel and long enough to cover the prepared segment with an additional 2–3 mm at either edge to minimise the risk of geographic miss (Figure 3a) [20]. The balloon manufacturer’s guidelines on maximum transit time in the patient and minimum inflation time should be followed. This is particularly important if the balloon inflation temporarily occludes a large branch (e.g., left main bifurcation), as prolonged ischemia may be poorly tolerated in patients with impaired ventricular function.

If the angiographic result in the main vessel is satisfactory after DCB application, the side branch should be evaluated. If flow is preserved and no worrying dissection is evident, no further intervention is required. Although carina shift is less common in the absence of a main vessel stent, plaque shift can compromise the side branch. In such cases, pre-dilation with a 1:1 balloon should be performed. If this restores TIMI III flow without significant dissection, a 1:1 DCB can then be applied. Routine kissing balloon inflation (KBI) is not mandated after a DCB-only strategy and may be best avoided to reduce the risk of drug washout. When necessary, KBI using a DCB is feasible with a 6 Fr guiding catheter [105]. Following the DCB-BIF study, KBI is generally considered acceptable following PCB use; however, its safety and efficacy after sirolimus-coated balloon (SCB) use remain uncertain [40]. In practice, KBI is best avoided when the DCB result is stable, considered when side branch flow or ostial geometry is suboptimal, and applied cautiously after SCB use given the limited supporting evidence.

If an optimal result cannot be achieved, owing to inadequate preparation (e.g., recoil) or the presence of a flow-limiting dissection, bailout with DES implantation is appropriate. Where only the main vessel requires stenting, a hybrid approach may be adopted; if the side branch is also compromised, any standard two-stent technique may be performed at the operator’s discretion

#### 7.1.3. Isolated Side Branch Disease

In isolated side branch lesions, both limbs of the bifurcation should be wired, and pre-dilation should be performed in the side branch using a 1:1 balloon. The balloon should extend slightly into the proximal main vessel to ensure adequate preparation of the ostium (Figure 3b). Owing to the fractal geometry of coronary bifurcations, this degree of protrusion is generally not expected to compromise the main vessel.

A DCB sized 1:1 may then be delivered, again with slight protrusion into the main vessel to ensure drug delivery to the ostium, provided the angiographic result after pre-dilation is satisfactory (Figure 3b). No treatment to the main vessel is required in this scenario.

If the result following DCB application is sub-optimal and bailout stenting is required following proximal optimisation, a KBI should be considered to open the stent struts into the main vessel. Given the high TLR associated with these lesions, conversion into a two-stent technique may be required.

#### 7.1.4. Two-DCB Strategy

In selected cases where treatment of both bifurcation limbs is indicated, each vessel should undergo thorough preparation with 1:1 balloons and, if necessary, plaque modification devices. If the angiographic result is satisfactory, a 1:1 DCB should first be applied to the side branch, followed by a 1:1 DCB in the distal main vessel extending into the proximal main vessel (Figure 3c).

The decision to use KBI should be based on the lesion/vessel result. KBI may be considered when there is evidence of significant carina shift or side branch compromise, whereas unnecessary inflation should be avoided to prevent disruption of the freshly delivered drug layer.

Although the two-DCB strategy has been described in small series and observational cohorts, robust randomised data supporting its broader use, particularly in the left main, are lacking. In contrast, randomised control trials such as DKCRUSH V and studies including EBC MAIN provide evidence favouring specific two-stent strategies in complex left main bifurcations [16,17,32]. Accordingly, a full-DCB approach in the left main should currently be regarded as a conceptual strategy rather than an evidence-based standard approach, and its use should ideally be restricted to clinical studies or highly selected cases with suitable anatomy.

If the main vessel result remains suboptimal despite DCB use, conversion to a hybrid strategy with DES implantation in the main vessel followed by proximal optimisation should be considered.

#### 7.1.5. Hybrid Strategy

A hybrid approach combining a DES in the main vessel with a DCB in the side branch may be appropriate in two scenarios.

(i) Upfront treatment of both branches.

In bifurcations where side branch treatment is anticipated and a two-stent technique would traditionally have been considered, both branches should be wired, imaged, and prepared with appropriate modification devices.

Limited data exist to guide the sequence of DCB side branch inflation. One option is to deliver the DCB to the side branch before main vessel stenting. This ensures full drug delivery to the side branch ostium and avoids the risk of the drug being stripped during recrossing. However, if plaque or carina shift occurs after main vessel stenting, further ballooning may be required in the side branch, potentially leading to drug washout. Bailout side branch stenting has been reported in approximately 10% of such cases [106].

If the side branch is treated first, the result following pre-dilation should be assessed. If satisfactory, in accordance with the previously described criteria, a 1:1 DCB long enough to cover the prepared segment and protrude 2–3 mm into the main vessel can be inflated. A DES can then be implanted in the main vessel (sized to the distal reference diameter) followed by proximal optimisation. KBI is not required unless there is significant carina or plaque shift. When KBI is needed, existing data support its use following PCB treatment, but data are lacking for its use following an SCB [40].

Alternatively, if the main vessel is treated first, a DES should be implanted, followed by proximal optimisation. The side branch is then rewired, the stent struts opened, and a DCB applied. Repeat proximal optimisation should follow. A third approach, described in a small series of 14 patients, involves inflating a jailed DCB in the side branch during main vessel stent implantation and maintaining inflation during post-dilatation followed by proximal optimisation; procedural success was 100% with no MACEs at the 12-month follow up [107].

(ii) Stepwise provisional strategy with subsequent side branch compromise.

In cases where an initial provisional strategy necessitates later side branch treatment, the branch should be rewired, the stent struts opened, and pre-dilation performed, often with KBI. If the angiographic result is satisfactory, a DCB may then be applied, followed by KBI and consideration of repeat proximal optimisation. If compromise persists, conversion to a two-stent technique should be considered.

## 8. Knowledge Gaps and Future Research Directions

DCBs represent a promising strategy for the treatment of coronary bifurcation lesions; however, the existing body of RCT data remains limited and generally underpowered to draw definitive conclusions on clinical endpoints. It is therefore imperative to evaluate the data in light of the specific patient and bifurcation factors in order to deliver the optimal procedural results.

Further large-scale, multi-centre RCTs are required to establish the comparative effectiveness of DCB-based strategies, including hybrid DCB-DES and DCB-only approaches, against contemporary two-stent techniques. This is particularly relevant in true bifurcation lesions and anatomically complex cases. High-risk subgroups, including patients with diabetes mellitus and those with complex bifurcation anatomy (e.g., Medina 1,1,1), remain significantly underrepresented in current clinical trials. Although observational data suggest that DCBs may confer advantages in these populations, such as reduced total stent length and the potential for shorter durations of dual antiplatelet therapy, these observations are largely extrapolated from ISR and small vessel data rather than bifurcation-specific RCTs [20,108,109]. Prospective, adequately powered RCTs with real-world inclusion criteria are needed to validate these findings and inform optimal treatment pathways in these challenging cohorts.

Ongoing studies, such as the Hybrid DEB, EBC-DCB, and PROVISION-DEB trials, are expected to provide meaningful insights, with endpoints including target lesion failure and long-term clinical outcomes assessed over three years (Table 3) [110,111]. The Hybrid DEB is a multi-centre, randomised study that will compare a hybrid DEB approach with a stepwise provisional two-stent strategy in patients with true bifurcation lesions [111]. Comparatively, the PROVISION-DEB clinical trial will compare a one-stent strategy with a drug-eluting balloon and a planned two-stent strategy on non-LM coronary true bifurcation with stratified diabetes [110]. Given most existing trials report follow-up data limited to 12–24 months, the long-term durability of DCB outcomes, particularly in relation to restenosis prevention, late lumen loss, and vessel remodelling, requires further review in larger patient populations with extended follow up.

While further data are awaited, DCB-based strategies do offer certain advantages over conventional stenting in a real-world setting. In routine practice, DCBs may be particularly useful in patients with small or diffusely diseased side branches, those for whom limiting stent burden is desirable, or cases where anatomy raises concern about carina shift or complex two-stent techniques. A hybrid approach with main vessel stenting and side branch DCB use remains the most practical and widely supported option, but full DCB strategies may be considered when lesion preparation is optimised alongside favourable vessel anatomy. Until more robust data are available, enrolment into ongoing clinical trials or registries should be encouraged whenever feasible.

Another key consideration for future research is the integration of intravascular imaging modalities, such as IVUS and OCT, into DCB-based strategies. Imaging guidance has gained a class 1 indication in recent guidelines for DES implantation during complex PCI. It is likely that intravascular imaging will enhance lesion preparation, optimise DCB delivery, and ensure adequate vessel sizing, factors particularly relevant in the context of bifurcation anatomy [112,113,114]. Nevertheless, at this stage there are insufficient data to provide intravascular imaging targets for optimal DCB PCI. Significant research is therefore required within bifurcation and non-bifurcation DCB PCI to both define the intravascular imaging goals and to assess whether the use of intravascular imaging provides improved outcomes in DCB PCI.

Finally, there are a broad range of DCB devices currently available, with substantially different designs, and as such it is likely that they will perform differently. The effect of DCBs should therefore not be considered a class effect but rather each should be considered on an individual basis. Further research is therefore needed to evaluate the performance of different DCBs in bifurcation disease. It would also be of significant interest to evaluate whether certain DCBs perform better in the main vessel or side branch.

## 9. Conclusions

DCBs have the potential to simplify complex bifurcation PCI procedures and may improve long-term outcomes in selected patients. Evidence is currently strongest for a hybrid approach using a DES in the main vessel and a DCB in the side branch, with meticulous lesion preparation essential for durable results. While the data thus far are promising, further specific bifurcation studies, with longer-term follow up, are needed. Lastly, the array of data on bifurcation management, much of which is discrepant, highlights the need to evaluate the various lesion- and patient-specific factors that will help guide the optimal treatment choice.

## Figures and Tables

**Table 1:** Summary of key clinical trials and studies on bifurcation lesion stenting.

Study/Trial	Year	N	Study Design	Inclusion Criteria	Primary Endpoint(s)	Follow-Up	Results	Limitations	Conclusion
**Traditional DES strategies**									
**DKCRUSH V [32]**	2017	482	Multicentre randomised trial DK crush two-stent (n = 240) vs. PS DES (n = 242)	True de novo distal LM bifurcation lesion (Medina 1,1,1 or 0,1,1) with >50% diameter stenosis of both the ostial LAD and LCx	TLF at 1 year	Angiographic at 13 months and clinical at 1, 7, and 12 months	TLF at 1 year: 5.0% DK crush vs. 10.7% PS (*p* = 0.02) Angiographic restenosis within LM complex: 7.1% vs. 14.6% (*p* = 0.10) Clinically driven TLR: 3.8% vs. 7.9% (*p* = 0.06)	Excluded non-true bifurcations Not powered for rare events Intravascular imaging not performed in >50% procedures FFR guidance of SB stenting in the PS group not routinely performed POT and KBI performed more often with DK crush than PS	DK crush 2-stent strategy superior to provisional DES for LM bifurcation
**DEFINITION II [33]**	2020	653	Multicentre randomised trial Two-stent group (n = 328) vs. PS approach (n = 325) (DEFINITION criteria-defined complex coronary bifurcation lesions)	Medina 1,1,1 or 0,1,1 with reference vessel diameter in the SB ≥2.5 mm, and had to be a complex bifurcation per DEFINITION criteria	TLF at 1 year	Angiographic at 13 months and clinical at 1, 6, and 12 months	TLF: 11.4% in PS vs. 6.1% in two-stent group (*p* = 0.019), largely driven by target vessel MI and clinically driven TLR in the PS group Cardiac death: 2.5% PS vs. 2.1% two-stent (*p* = 0.772)	Not DCB-focused, complex bifurcations only Intravascular imaging used in only 25% of patients	Favoured two stent technique for complex bifurcation lesions
**EBC MAIN [16]**	2021	467	Multicentre randomised trial Randomised patients with true LM bifurcations to dual stent (n = 237) vs. PS approach (n = 230)	True LM bifurcation (Medina 1,1,1 or 0,1,1) in which vessel reference diameters were ≥2.75 mm, suitable for PCI	Composite of all-cause death, myocardial infarction, and target lesion revascularisation at 12 months	Clinical at 6 and 12 months	Primary endpoint composite: 14.7% in PS vs. 17.7% in dual stent (*p* = 0.34) Death: 3.0% PS vs. 4.2% dual stent (*p* = 0.48) MI: 10.0% vs. 10.1% (*p* = 0.91) TLR: 6.1% vs. 9.3% (*p* = 0.16) Stent thrombosis: 1.7% vs. 1.3% (*p* = 0.90)	Open design Not DCB-focused Excluded non-true bifurcations European centres only	Fewer MACE with PS approach, however, nil statistically significant difference at 12 months between approaches.
**Isolated side branch disease**									
**PEPCAD-BIF [34]**	2016	64	Multicentre, RCT DCB-only in SB DCB (n = 32) vs. POBA (n = 32)	De novo bifurcation, side branch ≥2.0 mm, main branch DES	LLL at 9 months	Angiographic at 9 months	LLL 0.13 mm DCB vs. 0.51 mm POBA (*p* = 0.013) TLR: 1 patient in DCB, 3 patients in POBA Restenosis rate 6% DCB vs. 26% POBA (*p* = 0.045)	Small sample Surrogate endpoint Short follow-up	DCB superior to POBA for SB or distal main branch LLL
**Full DCB Treatment**									
**Schulz et al. [35]**	2014	39	Observational study Consecutive DCB only interventions in de novo bifurcation lesions with SB ≥2 mm	Bifurcation lesions with SB ≥2.0 mm, de novo lesions treated with DCB only	MACE: TLR, restenosis at 4 months	Angiographic at 4 months; patients refusing angiography had telephone follow-ups	Restenosis at 4 months: 10% TLR: 7.7%	Single centre Non-randomised Short follow-up	Treatment of de novo bifurcation lesions with DCB only intervention without additional stenting is a safe therapy
**Bruch et al. [36]**	2016	127	Single arm observational study DCB only (n = 70) vs. DCB + BMS (n = 57)	DCB for bifurcation	Clinically driven TLR at 9 months	Clinical at 9 months	TLR: 4.5% DCB-only vs. 3.6% DCB + BMS (*p* = 0.802) MACE: 6.1% vs. 7.3% (*p* = 0.789)	Small sample Non-randomised Short follow-up	DCB-only strategy is safe & effective for selected bifurcations
**Kitani et al. [37]**	2020	129	Retrospective, multicentre registry Combined DCA/DCB with follow-up angiogram	Major bifurcation lesion involving SB ≥2.0 mm in diameter De novo lesion DCA + DCB for bifurcation	TVF at 12 months	Angiographic at 6–15 months	TLR at 12 months: 3.1% TVF: 10.9% TVR: 1.6%	Retrospective Selection bias Small sample size Only paclitaxel-coated balloon was used as DCB	DCA/DCB provided good clinical outcomes and minimal SB damage and could be an optimal non-stent PCI strategy for bifurcation lesions
**Hybrid strategy**									
**BEYOND [38]**	2020	222	Prospective, multicentre RCT DES in MV, PCB in SB (n = 113) vs. DES in MV, BA in SB (n = 109)	De novo bifurcation lesion in the MV scheduled for DES, with SB scheduled for DCB, with reference vessel diameter ≥1.25 mm	Angiographic TLS at 9 months	Angiographic at 9 months and clinical at 30, 180, and 270 days after surgery	TLS: 28.7% PCB vs. 40.0% BA (*p* < 0.0001) LLL: −0.06 ± 0.32 PCB vs. 0.18 ± 0.34 mm BA (*p* < 0.0001) Nil TVR/TLR, TLF, all-cause death, cardiac death or thrombosis in either group	Short follow-up	In non-LM bifurcations treated with provisional T stenting, PCB in SB group demonstrated better angiographic results than treatment with regular BA
**HYPER trial sub-study [39]**	2023	50	Sub-study of the HYPER trial- a prospective, single-arm, multicentre, pilot Patients with true CBL were treated with a hybrid strategy (DCB and DES)	CBL involving the SB and at least one main vessel, i.e., 1,0,1; 0,1,1; or 1,1,1 lesions per Medina classification Involvement of a segment with a reference vessel diameter <2.75 mm.	Device-oriented composite endpoint (DOCE; including cardiac death, TV-MI, and ID-TLR) within 1 year	Clinical at 30 days, 6 months and 1 year	Procedural success was 96%, one case of peri-procedural myocardial infarction and one case of TLF (in a DES-treated segment) at 1 year	Small sample size Observational study No angiographic follow-up	Hybrid strategy combining DES and DCB was a feasible & safe option for treating CBL with a small calibre SB
**DCB-BIF [40]**	2025	784	Multicentre RCT DCB (n = 391) vs. NCB (n = 393) for SB after provisional stenting for true coronary bifurcation lesions	Simple and true CBL (Medina 1,1,1; 0,1,1; 1,0,1) Target lesions reference vessel diameter (both MV and SB) of ≥2.5 mm, baseline diameter stenosis of ≥50%, SB lesion length of <10 mm	MACE, a composite of cardiac death, target vessel myocardial infarction, or clinically driven target-lesion revascularisation at 1 year	Clinical +/- Angiographic at 1 year	MACE at 1 year: 7.2% DCB vs. 12.5% NCB (*p* = 0.013)	Potential performance bias (random assignment to DCB and NCB angioplasty for SB not masked) Complex bifurcation lesions excluded Sirolimus-coated balloons not used Intravascular imaging guidance not used for all patients, albeit similarly used in two groups Fewer female participants (23.2%) Interventional procedures not guided by physiologic assessment	For provisional stenting, MV stenting with a DCB for the compromised SB resulted in a lower 1-year MACE rate compared with an NCB SB intervention
**REC-CAGEFREE I [41]**	2024	2272	Open-label randomised, non-inferiority trial Paclitaxel-coated balloon/DCB angioplasty (n = 1133) with the option of rescue stenting vs. DES (n = 1139) Bifurcation lesions (n = 773); bifurcation involved in 32.5% lesions in DCB group, 31.1% DES	De novo, non-complex target lesions, any vessel diameter	Device-oriented composite endpoint (DOCE; including cardiovascular death, TVMI, and TLR) at 24 months	Clinical at 1 (±14 days), 3, 6, 12, 18, and 24 (±30 days) months after randomisation	24-month DOCE: 6.4% DCB vs. 3.4% DES (*p* = 0.0008); criterion for non-inferiority not met 9.4% in DCB received rescue DES after unsatisfactory DCB angioplasty Periprocedural MI: 0.9% DCB vs. 0.8% DES Equipoise in outcomes for bifurcation lesion subgroup: HR 1.25, 95%CI 0.70–2.23	Excluded complex/bifurcation lesions Open-label Short follow-up Patients only treated with paclitaxel-coated balloons Study only conducted in China with an east Asian population	DCB with rescue stenting did not achieve non-inferiority vs. intended DES implantation in terms of the DOCE at 2 years, indicating DES as preferred treatment.
**SELUTION DeNovo [42]**	2025	3326	Randomised, multicentre, international, single-blind, clinical trial Comparing a PCI strategy with sirolimus eluting balloon and provisional DES or systematic DES	PCI indicated for ≥1 lesion considered suitable for treatment by either sirolimus eluting balloon or DES and clinical presentation with chronic coronary syndrome, unstable angina or non-ST segment elevation myocardial infarction Target lesions diameters between 2 and 5 mm.	Target vessel failure (TVF) at 1 and 5 years (composite endpoint comprised of cardiac death, target vessel myocardial infarction or clinically driven target vessel revascularisation)	Clinical follow-up at 30 days, 6 months, 1, 2, 3, 4, and 5 years	12 month TVF in DES 4.4% vs. 5.3% in DCB (*p* = 0.02 for variable non-inferiority margin)	Manuscript not currently available for full review	Selution DCB is a reasonable alternative to DES in denovo coronary disease (excluding LMS, STEMI, CTO, grafts)

DCB: drug-coated balloon; DES: drug-eluting stent; SB: side branch; MACE: major adverse cardiovascular events; TLR: target lesion revascularisation; TVR: target vessel revascularisation; POBA: plain old balloon angioplasty; LLL: late lumen loss; BMS: bare metal stent; DK: double kissing; TLF: target lesion failure; LM: left main; MI: myocardial infarction; CBL: coronary bifurcation lesions; MV: main vessel; PCB: paclitaxel-coated balloon; BA: balloon angioplasty; TLS: target lesion stenosis; NCB: non-compliant balloon; PS: provisional stenting; DCA: directional coronary atherectomy; ID-TLR: ischaemia-driven TLR; TVMI: target vessel myocardial infarction.

**Table 2:** Comparison of Antiproliferative Agents in Drug-Coated Balloons.

Parameter	Paclitaxel-Coated Balloon (PCB)	Sirolimus-Coated Balloon (SCB)
**Mechanism of Action**	Microtubule inhibitor (cytotoxic) Irreversible binding to β-tubulin, inhibits microtubule disassembly, halts cell cycle progression–cytotoxic	mTOR inhibitor (cytostatic) Binds FKBP-12, inhibits mTOR, blocks cell cycle progression G1→S, cytostatic effect
**Pharmacokinetics**	Highly lipophilic Rapid cellular uptake and longer tissue retention, irreversible	Less lipophilic Slower cellular uptake, reversible Requires encapsulation into nanocarriers
**Typical Dosage (μg/mm^2^)**	3	4
**Downstream Myocardial Injury**	More frequent (myocyte necrosis/scarring) due to narrower therapeutic range	Less frequent Wider safety therapeutic range
**Drug Concentration (Tissue)**	Higher	Lower
**Therapeutic range**	Wide	Narrow

**Table 3:** Ongoing clinical trials on drug-coated balloons.

Trial Identifier (ClinicalTrials.gov)	Status	Study Title	Study Design	Inclusion Criteria	Primary Endpoint(s)	Target Sample Size	Follow-Up	Completion Timeline
**Drug-coated balloons (DCB) in bifurcations**								
**NCT05731687**	Recruiting	Bifurcation PCI With a Hybrid Strategy With Drug Eluting Balloons Versus a Stepwise Provisional Two-stent Strategy (Hybrid DEB)	Multicentre randomised controlled trial and registry Compare hybrid DEB approach with a stepwise provisional two-stent strategy	De novo true coronary bifurcation lesions, left main (LM) and non-LM	Composite endpoint of the occurrence of all-cause death, periprocedural or spontaneous myocardial infarction and/or target vessel revascularisation (TLR)	500	Clinical at 12 months, and at median 2-year follow-up	Study start (actual): 2023-03 Estimated primary completion: 2026-03 Estimated study completion: 2030-03
**NCT06002932**	Recruiting	Comparison of PROVISIONal 1-stent Strategy with Drug-Eluting Balloon Versus Planned 2-stent Strategy in Patients with Non-LM Coronary True-Bifurcation Lesions (PROVISION-DEB)	Multicentre randomised trial One-stent strategy with DCB vs. planned two-stent strategy	Non-LM coronary true bifurcation lesions undergoing PCI	TLF (composite outcome of cardiac death, target vessel myocardial infarction or TLR)	750 stratified randomisation by diabetes, n = 375 in conventional arm (planned two-stent strategy) and N = 375 in PROVISION-DCB arm (provisional one-stent + DEB strategy)	3 years clinical follow-up (6, 12, and 36 months) Coronary angiography at discretion of investigator, typically at 6 months	Study start (actual): 2023-09 Estimated primary completion: 2028-07 Estimated study completion: 2028-12
**NCT06822322**	Recruiting	The European Bifurcation Club Randomized Trial of Stepwise Provisional Stenting Versus Drug Coated Balloon Therapy for Non-left Main True Coronary Bifurcations—EBC DCB Study	Prospective, multi-centre, open-label, randomised (1:1) non-inferiority trial	Non-LM bifurcations requiring revascularisation and both MV and SB diseased (≥2.5 mm and Medina 1,1,1; 1,0,1 or 0,1,1)	Bifurcation Orientated Composite Endpoint (BOCE): defined by cardiovascular death, target bifurcation-related myocardial infarction or target bifurcation revascularisation	750	Clinical follow-up at 6 months, 1 year, 3 years, 5 years, and 8 years	Study start (estimated): 2025-05 Estimated primary completion: 2028-05 Estimated study completion: 2035-08
**Intravascular lithotripsy with DCB**								
**NCT05625997**	Recruiting	Shockwave IVL + DCB	Observational, prospective	Coronary artery disease with PCI to treat lesions with criteria: de novo lesion with diameter stenosis >50% Coronary vessel diameter <3.0 mm Severe calcification at the target segment, defined as fluoroscopic radiopacities noted without cardiac motion prior to contrast injection involving both sides of the arterial wall in at least 1 location OR Intravascular Ultrasound/Optical Coherence Tomography demonstrated calcium angle of ≥270° on at least 1 cross section.	TLF, successful IVL + DCB (composite of IVL application at target site, DCB inflation, residual in-segment diameter stenosis <40% by quantitative coronary angiography, and no need for bailout stenting	50	1-month follow-up	Study start (actual): 2023-06 Estimated primary completion: 2025-06 Estimated study completion: 2026-06

## Data Availability

No new data were created or analyzed in this study. Data sharing is not applicable to this article.

## Abbreviations

The following abbreviations are used in this manuscript:

DCB	Drug-coated balloon
DES	Drug-eluting stent
ISR	In-stent restenosis
KBI	Kissing balloon inflation
LLL	Late lumen loss
PCI	Percutaneous coronary intervention
POBA	Plain old balloon angioplasty
POT	Proximal optimisation technique
